# Accuracy of High-Throughput Nanofluidic PCR-Based Pneumococcal Serotyping and Quantification Assays Using Sputum Samples for Diagnosing Vaccine Serotype Pneumococcal Pneumonia: Analyses by Composite Diagnostic Standards and Bayesian Latent Class Models

**DOI:** 10.1128/JCM.01874-17

**Published:** 2018-04-25

**Authors:** Satoshi Kakiuchi, Motoi Suzuki, Bhim Gopal Dhoubhadel, Akitsugu Furumoto, Hiroyuki Ito, Kei Matsuki, Yoshiko Tsuchihashi, Norichika Asoh, Michio Yasunami, Koya Ariyoshi, Konosuke Morimoto

**Affiliations:** a Department of Clinical Medicine, Institute of Tropical Medicine, Nagasaki University, Nagasaki, Japan; b Graduate School of Biomedical Sciences, Nagasaki University, Nagasaki, Japan; c School of Tropical Medicine and Global Health, Nagasaki University, Nagasaki, Japan; d Department of Internal Medicine, Juzenkai Hospital, Nagasaki, Japan

**Keywords:** Bayesian latent class models, Streptococcus pneumoniae, adult pneumonia, sputum culture, sputum real-time PCR, urinary antigen detection

## Abstract

The lack of reliable diagnostic tests for detecting vaccine serotype pneumococcal pneumonia (VTPP) remains a challenging issue in pneumococcal vaccine studies. This study assessed the performances of high-throughput nanofluidic PCR-based pneumococcal serotyping and quantification assay methods using sputum samples (the nanofluidic sputum quantitative PCR [Sp-qPCR] assay) to diagnose 13-valent pneumococcal conjugate VTPP compared with the performance of the serotype-specific urinary antigen detection (UAD) assay using urine samples. Adult pneumonia patients from Japan were enrolled in this study between September 2012 and August 2014. Sputum samples were subjected to the nanofluidic Sp-qPCR assay, quantitatively cultured, and serotyped by the Quellung reaction (SpQt). Urine samples were tested by the UAD method. The diagnostic performances of these tests were assessed using composite reference standards and Bayesian latent class models (BLCMs). Among 244 total patients, 27 (11.1%) tested positive with the UAD assay, while 16 (6.6%) and 34 (13.9%) tested positive with the SpQt and nanofluidic Sp-qPCR assays, respectively, with a cutoff value of ≥10^4^ DNA copies/ml, which showed the maximum value of the Youden index. Using BLCMs, the estimated prevalence for VTPP was 12.9%, and the nanofluidic Sp-qPCR assay demonstrated the best performance (sensitivity, 90.2%; specificity, 96.9%), followed by UAD (sensitivity, 75.6%; specificity, 97.9%) and SpQt (sensitivity, 45.8%; specificity, 99.5%). However, when a higher cutoff value of ≥10^7^ DNA copies/ml was applied, the performance of UAD became comparable to that of Sp-qPCR. The vaccine serotype-specific pneumococcal DNA load in sputum among UAD-positive patients was 3 logs higher than that among UAD-negative patients (*P* = 0.036). The nanofluidic Sp-qPCR assay may be accurate and useful for detecting VTPP among adults.

## INTRODUCTION

Streptococcus pneumoniae, or the pneumococcus, is the leading bacterial cause of morbidity and mortality among adults (1–4). Invasive pneumococcal disease (IPD) is the most severe form of pneumococcal infection, while pneumonia is the most common pneumococcal disease in this age group. More than 90 pneumococcal serotypes have been identified, and they vary in their virulence and transmissibility (5–7). Currently, two types of polyvalent pneumococcal vaccines are available for adults, the 23-valent pneumococcal polysaccharide vaccine (PPSV23) and the 13-valent pneumococcal polysaccharide conjugate vaccine (PCV13). Although these two vaccines demonstrate moderate to high protective efficacy against invasive pneumococcal disease (8–10), their efficacy and effectiveness against adult pneumococcal pneumonia (PP) vary by study (11–13).

The lack of reliable diagnostic tests for vaccine serotype pneumococcal pneumonia (VTPP) remains a challenging issue in pneumococcal vaccine studies. Blood cultures are considered a reference standard for diagnosing pneumococcal bacteremia, and the proportion of bacteremia cases among PP cases is only 5 to 20% (14, 15). A urinary capsule antigen immunochromatographic test (ICT; BinaxNOW S. pneumoniae; Alere, USA) is sufficiently specific (97.2%) but less sensitive (74.0%) according to a meta-analysis, but its test performance substantially varies by study because of its use of different reference standards (i.e., cultures of blood, sputum, pleural fluid, and bronchoalveolar lavage fluid) (16). Moreover, the ICT does not differentiate pneumococcal serotypes. Recently, a serotype-specific urinary antigen detection (UAD) assay was developed and used in a PCV13 trial conducted in the Netherlands (17). This multiplex immunoassay can detect in urine samples 13 capsular polysaccharides corresponding to the PCV13 serotypes. Although this assay demonstrated a high sensitivity (97.1%) and specificity (100%) for the detection of bacteremia (17), its performance regarding the detection of VTPP remains to be established.

We recently established a high-throughput pneumococcal serotyping and quantification method based on a nanofluidic real-time PCR assay and applied this method to pneumonia studies using sputum samples (the nanofluidic sputum quantitative PCR [Sp-qPCR] assay) (18–20). A few studies have investigated the accuracy of a PCR-based method using sputum samples for diagnosing PP, but its performance varied by study (sensitivity and specificity, 77.8 to 94.0% and 66.7 to 96.0%, respectively) (21, 22). These studies used composite diagnostics (i.e., a combination of blood cultures, sputum cultures, and ICT) as the reference standard, but none of these tests were perfect; thus, the performance of PCR-based methods may be underestimated. No study has investigated the implications of serotype-specific pneumococcal DNA loads in respiratory samples in diagnosing PP.

We conducted this study to establish the accuracy and usefulness of a high-throughput PCR-based pneumococcal serotyping and quantification method using sputum samples (Sp-qPCR) for diagnosing VTPP caused by a PCV13 type in comparison with those of other methods, including UAD. Test performance was evaluated by the conventional method using a composite reference standard and Bayesian latent class models (BLCMs), assuming an imperfect gold standard (23).

## MATERIALS AND METHODS

### Study patients and outline.

This study was conducted as part of a prospective study for adult pneumonia in Japan by the Adult Pneumonia Study Group—Japan (20) at Juzenkai Hospital, a community-based hospital located in the center of Nagasaki, Japan, from 10 September 2012 to 22 August 2014. Patients who fulfilled the following criteria were enrolled in the study: (i) an age of ≥15 years, (ii) the presence of symptoms compatible with pneumonia (e.g., fever, cough, sputum, pleuritic chest pain, and dyspnea), and (iii) the presence of new infiltrates on chest radiography or computed tomography scan films. Patients who developed pneumonia after 48 h of admission were excluded.

### Diagnostic tests.

Sputum and urine samples were collected from the enrolled patients upon their first hospital visit or admission. Quantitative sputum culture (SpCx) was performed at the hospital laboratory using the conventional method. According to a previous report (24), we used 1 × 10^5^ CFU/ml as the cutoff value for diagnosing PP. Pneumococcal isolates were transported to the Institute of Tropical Medicine at Nagasaki University for serotyping by the capsular Quellung reaction (SpQt) using antisera from the Statens Serum Institut (Denmark). Sputum samples were further tested by an in-house qPCR for *lytA* for pneumococcal detection (18) and by an in-house nanofluidic Sp-qPCR. Details of the methods and primers used were described previously (18, 19). In brief, DNA was extracted from sputum samples using a QIAamp DNA minikit (Qiagen) according to the manufacturer's instructions. All samples were screened by qPCR for the *lytA* gene to identify the presence of the pneumococcus (25). The *lytA*-positive samples (corresponding to ≥10^3^ DNA copies/ml) were subjected to the nanofluidic Sp-qPCR assay for detecting 29 serogroups/50 serotypes and measuring their pneumococcal DNA loads.

In the current study, a common primer set was used to identify serotypes 6A and 6B in our PCR-based serotyping system. Moreover, the UAD assay cannot discriminate serotypes 6C and 6D from serotypes 6A and 6B, respectively (17). According to previous studies, cross-immunological reactions have been observed between serotypes 6A and 6C and between serotypes 6B and 6D (26–28). We therefore grouped these serotypes into serogroup 6 and included them as PCV13 serotypes. We also grouped serotypes 18A, 18B, 18C, and 18F into serogroup 18 and included them as PCV13 serotypes.

The presence of the pneumococcal urinary antigen in urine samples was tested by ICT, and all urine samples were transported to Pfizer Vaccine Research (Pearl River, NY, USA) for the UAD assay. Details regarding the assay were described previously (17). Prior to the current study, UAD assay cutoffs were updated from the original cutoffs established according to the results for 400 control samples collected in the United States (29). To validate the updated cutoffs for Japanese patients, we collected 202 urine samples from age-matched afebrile inpatients and outpatients at our study site, and only 1 sample showed a positive result (false-positive rate, 0.5%). Therefore, the updated cutoffs were applied to all samples in the current study.

### Analysis.

The characteristics of the enrolled patients were described using a simple tabulation. The prevalence of PP and VTPP (i.e., PP caused by serotypes 1, 3, 4, 5, 7F, 9V, 14, 19A, 19F, and 23F and serogroups 6 and 18) determined by diagnostic tests was summarized. Two approaches were used to estimate the diagnostic accuracies of the qPCR for *lytA* for PP and the nanofluidic Sp-qPCR assay for VTPP. We first considered (i) the urinary antigen test (ICT for PP, UAD for VTPP) and (ii) composite diagnostics (a combination of ICT and SpCx for PP, a combination of UAD and SpQt for VTPP) as the reference standard. Then, we estimated the accuracies of all tests using BLCMs (30). BLCMs do not assume that any test is perfect and estimate the accuracy of each test on the basis of the observed frequency of the possible combinations of test results. The models assumed that a correlation existed between the sputum cultures and both the qPCR assay for *lytA* and the nanofluidic Sp-qPCR assay. The mean values of disease prevalence, sensitivity, and specificity with 95% credible intervals (CrI) were determined using the WinBUGS (version 1.4) program (31). In this analysis, we focused each test identification result on PCV13 serotypes and not on the concordance of the serotype identification result of each test. Different cutoff values were examined to evaluate the accuracy of the qPCR assay for *lytA* and the nanofluidic Sp-qPCR assay. We used receiver operating characteristic analysis and the Youden index (sensitivity + specificity − 1) to explore the optimal cutoff values for the qPCR for *lytA* and the nanofluidic Sp-qPCR assay (32). The point that gave the maximum Youden index value was considered the optimal cutoff (33).

### Ethics.

This study was conducted in accordance with the Guidelines for Ethical Aspects in Epidemiological Study (Ministry of Health, Labor and Welfare of Japan, 2008) and was approved by the Institutional Review Board (IRB) of the Institute of Tropical Medicine at Nagasaki University and the IRB of Juzenkai Hospital. The requirement for obtaining written consent from all participants was waived by both IRBs because of the study's observational nature with no deviation from the current medical practice. Anonymized data were used for our analyses.

## RESULTS

### Patients' backgrounds.

During the study period, a total of 288 pneumonia patients were enrolled, among which 44 were excluded from the study, 39 were not tested by sputum culturing, and 5 were not tested by the nanofluidic Sp-qPCR. Finally, 244 were eligible for analysis (see Fig. S1 in the supplemental material).

The baseline characteristics of the study patients are shown in Table 1. In total, 151 (62%) participants were male, and most (70.5%) were 75 years old or older, with the median age being 80 years. A total of 147 (60%) patients were classified as having community-acquired pneumonia, and the remaining patients were classified as having health care-associated pneumonia. Twenty (8%) patients had received PPSV23, while none had received PCV13. Sixty (25%) patients were prescribed antibiotics before enrollment, and 11 (5%) patients died before discharge.

**TABLE 1:** Characteristics of the study population^*a*^

Characteristic	Values
Median (range) age (yr)	80 (22–99)
No. (%) of male patients	151 (61.9)
No. (%) of patients who received PPSV23	20 (8.2)
No. (%) of patients with community-acquired pneumonia	147 (60.3)
No. (%) of inpatients	226 (92.6)
No. (%) of patients with the following underlying disease:	
Diabetes	47 (19.3)
Hypertension	113 (46.3)
Hyperlipidemia	22 (9.0)
Asthma	24 (9.8)
COPD^*b*^	63 (25.8)
No. (%) of patients who used antibiotics before enrollment	60 (24.6)
No. (%) of patients who died	11 (4.5)

a Data are for a total of 244 patients.

b COPD, chronic obstructive pulmonary disease.

### Overall pneumococcus-positive rates.

Among the 244 patients, 30 (12.3%) tested positive by ICT. The pneumococcus-positive rates differed by the cutoff values for the detection of pneumococcus by SpCx and *lytA* by qPCR; 3.3% to 11.5% were positive for pneumococcus by SpCx, and 2.5% to 21.7% were positive for *lytA* by qPCR (Table S1). Blood samples for culture were collected from 58 patients (23.8%), and one yielded S. pneumoniae (1.7% of the tested patients). The prescription of antibiotics did not change the pneumococcus-positive rates.

The prevalence of VTPP was 11.1%, based on UAD results, and it varied from 2.5% to 6.6%, based on SpQt, according to the cutoff value. When the nanofluidic Sp-qPCR assay was applied, the VTPP prevalence ranged widely from 3.3% to 14.8%, according to the cutoff value (Table S2). The prescription of antibiotics was also not associated with the rates of positivity for a vaccine serotype pneumococcus except when the cutoff value of 1.0 × 10^9^ DNA copies/ml was used for Sp-qPCR. The most frequent vaccine serotype was serotype 3, followed by 6 and 19A, regardless of the method (Table S3). Among 53 patients who tested positive for any pneumococcal serotype by the nanofluidic Sp-qPCR (cutoff value, 1.0 × 10^4^ DNA copies/ml), 25 (47%) tested positive for multiple serotypes and 11 (21%) tested positive for three serotypes. Serotypes 4 and 5 were detected more frequently by the nanofluidic Sp-qPCR than by the other tests. All serotypes identified by SpQt were compatible with the dominant serotypes identified by the nanofluidic Sp-qPCR, except in two samples (samples 32 and 50). The serotypes identified by UAD were also compatible with those identified by nanofluidic Sp-qPCR, except in two samples which were positive for serotype 23F by UAD (samples 25 and 52).

### Diagnostic accuracy of nanofluidic Sp-qPCR, UAD, and SpQt.

The disease prevalence in the study population as well as the sensitivity and specificity of the nanofluidic Sp-qPCR assay with different cutoffs for VTPP were estimated according to different reference standards (Table 2). The disease prevalence based on the combined results of UAD and SpQt (cutoff value, 1 × 10^5^ CFU/ml) was 12.3%. The Youden index demonstrated a maximum value of 1.0 × 10^4^ DNA copies/ml regardless of the reference standard. When this value was used as the cutoff for the nanofluidic Sp-qPCR, the sensitivity and specificity were 81.5% (95% confidence interval [CI], 61.9 to 93.7%) and 94.5% (95% CI, 90.5 to 97.1%), respectively, in comparison with the results of UAD; they were 83.3% (95% CI, 65.3 to 94.4%) and 95.9% (95% CI, 92.2 to 98.1%), respectively, using composite diagnostics as the reference. When the higher cutoff value was used, the sensitivity became lower and the specificity became higher.

**TABLE 2:** Estimated disease prevalence and sensitivity and specificity of using serotype-specific qPCR to diagnose VTPP^*a*^

Serotype-specific pneumococcal DNA load cutoff value (no. of copies/ml)	UAD assay				UAD assay + sputum culture^*b*^			
	Disease prevalence (%)^*c*^	Sensitivity (%)	Specificity (%)	Youden index	Disease prevalence (%)	Sensitivity (%)	Specificity (%)	Youden index
≥10^3^	11.1 (7.4–15.7)	81.5 (61.9–93.7)	93.5 (89.4–96.4)	0.75	12.3 (8.4–17.1)	83.3 (65.3–94.4)	94.9 (91.0–97.4)	0.782
≥10^4^		81.5 (61.9–93.7)	94.5 (90.5–97.1)	0.76		83.3 (65.3–94.4)	95.9 (92.2–98.1)	0.792
≥10^5^		74.1 (53.7–88.9)	95.4 (91.7–97.8)	0.695		76.7 (57.7–90.1)	96.7 (93.4–98.7)	0.734
≥10^6^		70.4 (49.8–86.2)	97.7 (94.7–99.2)	0.681		70.0 (50.6–85.3)	98.6 (96.0–99.7)	0.686
≥10^7^		63.0 (42.4–80.6)	98.2 (95.3–99.5)	0.612		63.3 (43.9–80.1)	99.1 (96.6–99.9)	0.624
≥10^8^		51.9 (31.9–71.3)	99.1 (96.7–99.9)	0.51		46.7 (28.3–65.7)	99.1 (96.6–99.9)	0.458
≥10^9^		22.2 (8.6–42.3)	99.1 (96.7–99.9)	0.213		20.0 (7.7–38.6)	99.1 (96.7–99.9)	0.191

a qPCR, quantitative PCR; UAD, serotype-specific urinary antigen detection assay. Values in parentheses are 95% confidence intervals.

b Determined by conventional sputum culture and by use of the Quellung reaction's bacterial load cutoff value of 1.0 × 10^5^ CFU/ml.

c Estimated from the reference standard or the composite reference standard result assuming 100% specificity.

We then estimated the accuracies of all tests using BLCMs (Table 3). When the cutoff value of 1.0 × 10^4^ DNA copies/ml with the maximum Youden index was used, the nanofluidic Sp-qPCR was the most sensitive test for VTPP (sensitivity, 90.2%; 95% credible interval [CrI], 71.2 to 99.7%), followed by UAD (sensitivity, 75.6%; 95% CrI, 55.4 to 92.4%) and SpQt (sensitivity, 45.8%; 95% CrI, 27.5 to 65.8%), while the three tests showed comparable specificities (96.9% [95% CrI, 93.4 to 99.7%], 97.9% [95% CrI, 95.1 to 99.9%], and 99.5% [95% CrI, 98.2 to 100%], respectively). Notably, the accuracy of UAD was almost comparable to that of the nanofluidic Sp-qPCR when the cutoff value was 1.0 × 10^7^ DNA copies/ml or higher.

**TABLE 3:** Estimated disease prevalence and sensitivity and specificity of using serotype-specific qPCR, sputum culture, and UAD to diagnose VTPP by BLCM analysis^*a*^

Serotype-specific pneumococcal DNA load cutoff value (no. of copies/ml)	Disease prevalence	Serotype-specific qPCR		Quellung reaction^*b*^		UAD		Youden index of PCR
		Sensitivity (%)	Specificity (%)	Sensitivity (%)	Specificity (%)	Sensitivity (%)	Specificity (%)	
≥10^3^	13.0 (8.3–18.8)	89.9 (71.5–99.7)	96.1 (92.2–99.4)	45.2 (26.9–65.1)	99.5 (98.2–100)	75.0 (54.1–92.4)	97.9 (95.0–99.9)	0.860
≥10^4^	12.9 (8.3–18.5)	90.2 (71.2–99.7)	96.9 (93.4–99.7)	45.8 (27.5–65.8)	99.5 (98.2–100)	75.6 (55.4–92.4)	97.9 (95.1–99.9)	0.871
≥10^5^	12.1 (7.5–17.8)	87.7 (63.9–99.7)	97.5 (94.3–99.8)	49.0 (28.7–70.2)	99.5 (98.3–100)	75.6 (55.1–92.3)	97.2 (94.0–99.8)	0.852
≥10^6^	10.7 (6.5–16.0)	86.0 (61.4–99.6)	98.7 (96.5–99.9)	51.4 (30.7–72.6)	99.1 (97.5–99.9)	82.0 (62.9–95.6)	97.0 (93.7–99.9)	0.847
≥10^7^	9.9 (5.8–15.3)	84.0 (55.4–99.6)	99.0 (97.0–100)	56.0 (32.7–78.2)	99.1 (97.5–99.9)	81.7 (62.3–95.7)	96.3 (92.6–99.6)	0.830
≥10^8^	7.7 (3.9–13.4)	78.5 (43.2–99.3)	98.7 (96.8–99.9)	61.4 (31.6–87.6)	98.3 (96.2–99.6)	91.7 (72.4–99.8)	95.3 (91.2–99.5)	0.772
≥10^9^	7.7 (3.4–14.3)	35.6 (13.8–64.6)	98.6 (96.7–99.8)	67.8 (33.6–97.5)	98.5 (96.3–99.9)	86.9 (61.7–99.6)	94.9 (90.4–99.4)	0.342

a qPCR, quantitative PCR; UAD, serotype-specific urinary antigen detection assay; BLCM, Bayesian latent class model. Values in parentheses are 95% credible intervals.

b Determined by conventional sputum culture and by use of the Quellung reaction's bacterial load cutoff value of 1.0 × 10^5^ CFU/ml.

### Serotype-specific pneumococcal DNA load.

Serotype-specific pneumococcal DNA loads were compared by the UAD result statuses (Fig. S2). The median pneumococcal serotype-specific DNA load in UAD-positive patients (1.1 × 10^8^ DNA copies/ml) was substantially higher than that in UAD-negative patients (1.2 × 10^5^ DNA copies/ml) (*P* = 0.036, Wilcoxon rank-sum test).

### Diagnosing pneumococcal pneumonia.

We also investigated the performances of assaying sputum for pneumococcal DNA (*lytA*) for diagnosing pneumococcal pneumonia in comparison with those of SpCx and ICT. The cutoff value for the qPCR detection of *lytA* determined from the maximum Youden index was also 1.0 × 10^4^ DNA copies/ml. When composite diagnostics (ICT and SpCx) were used as the reference standard, the sensitivity and specificity of using qPCR to detect *lytA* were 85.4% (95% CI, 70.8 to 94.4%) and 94.6% (95% CI, 90.5 to 97.3%), respectively (Table S4). When we estimated the accuracy of these tests using BLCMs, the prevalence was 18.3%, and using qPCR to detect *lytA* was the most sensitive for the detection of PP (sensitivity, 91.4%; 95% CrI, 74.5 to 99.8%), followed by ICT (sensitivity, 60.0%; 95% CrI, 43.2 to 76.2%) and SpCx (sensitivity, 56.7%; 95% CrI, 39.4 to 74.2%); these tests also showed comparable specificities (97.0% [95% CrI, 92.7 to 99.8%], 97.8% [95% CrI, 94.8 to 99.8%], and 99.1% [95% CrI, 97.3 to 99.9%], respectively) (Table S5). The median pneumococcal DNA load in patients deemed positive by both ICT and qPCR for *lytA* (4.7 × 10^7^ DNA copies/ml) was also substantially higher than that in patients deemed negative by ICT and positive by qPCR for *lytA* (2.6 × 10^6^ DNA copies/ml) (*P* = 0.005, Wilcoxon rank-sum test).

## DISCUSSION

The nanofluidic Sp-qPCR assay with a cutoff value of 1.0 × 10^4^ DNA copies/ml demonstrated the highest degree of accuracy for diagnosing VTPP compared with UAD and conventional culture-based methods in the BLCM analysis; its sensitivity and specificity were 90.2% and 96.9%, respectively.

None of the existing diagnostic tests for VTPP and PP, such as blood culture, sputum culture, and ICT, are perfect; thus, composite reference standards using these tests are inaccurate. Using imperfect composite reference standards may underestimate the true accuracy of new tests (34). To overcome this limitation, we also estimated the accuracy of PCR-based methods using BLCMs. BLCMs have been used to evaluate the accuracy of tests when no reference standard tests exist (23, 30, 35). In the current study, the estimated sensitivities and specificities of the nanofluidic Sp-qPCR and qPCR assays for *lytA* were higher than those of sputum culture and urinary antigen tests. Our findings suggest that PCR-based methods (nanofluidic Sp-qPCR and qPCR for *lytA*) may be highly accurate in diagnosing VTPP and PP among adults.

The capsular Quellung test is a conventional method for pneumococcal serotyping and has been used as a gold standard for an extended period. However, this method is labor-intensive and time-consuming. Moreover, because this method requires pneumococcal isolates, its sensitivity is limited by that of the culture method. To conduct PCV13 vaccine efficacy surveys, the UAD assay was developed to diagnose VTPP. A validation study demonstrated good performance by the UAD assay (sensitivity, 97.1%; specificity, 100%); however, these values were estimated using a bacterial culture as the reference (17). In the current study, the UAD assay demonstrated a high specificity (97.6%) but a relatively low sensitivity (75.6%) for diagnosing VTPP when a cutoff of 1.0 × 10^4^ DNA copies/ml was applied for the nanofluidic Sp-qPCR. Furthermore, the median sputum serotype-specific pneumococcal DNA load in the UAD-positive group was nearly 3 logs higher than that in the UAD-negative group. This result indicates that UAD can possibly identify VTPP with a relatively large amount of sputum pneumococcal serotype-specific DNA among adults. In fact, when the serotype-specific pneumococcal DNA cutoff value was 1.0 × 10^7^ DNA copies/ml, the nanofluidic Sp-qPCR and UAD diagnostic accuracies were estimated to be at nearly the same level (sensitivities, 84.0% and 81.7%, respectively; specificities, 99.0% and 96.3%, respectively).

Although most serotypes identified by the nanofluidic Sp-qPCR, SpQt, and UAD assays were identical, some discordant findings were observed. Serotypes 23F and 1 were more frequently detected by UAD than by the nanofluidic Sp-qPCR or SpQt (see Table S3 in the supplemental material). Considering the low detection rate in sputum samples, these serotypes might have been causing bacteremia without pneumonia among our cases. In one case (case 25), serotype 22F was detected by the nanofluidic Sp-qPCR and SpQt and serotype 23F was detected by UAD. This finding might suggest that the focus of serotype 22F infection was limited to the respiratory tract, while that of 23F was outside the respiratory tract in this case. Further serotype-specific verification may be necessary.

The current study demonstrated an additional advantage of the nanofluidic Sp-qPCR assay, as it was capable of detecting multiple serotypes. The presence of multiple pneumococcal serotypes is difficult to recognize with conventional culture and the Quellung reaction methods, especially because the quantity of the second most dominant serotype is, on average, 2 logs less than that of the most dominant serotype (36–38). In our study, multiple serotypes were detected in approximately half of the adult PP patients by the nanofluidic Sp-qPCR, but none of them were detected by the conventional culture-based method. UAD also had a capacity to detect multiple serotypes; one case was positive for both serogroups 6 and 18 by UAD in our study. We could not elucidate the clinical and epidemiological meanings of detection of multiple serotypes or the associations of secondary and tertiary serotypes with the primary serotype in adult PP patients because of the small sample size. Further studies are needed to establish the clinical implications of multiple serotypes.

We also evaluated the feasibility of detecting *lytA* by qPCR to diagnose PP. A few studies have evaluated the diagnostic accuracy of PCR-based assays for PP using respiratory samples and demonstrated inconsistent findings. Stralin et al. showed that the sensitivity and specificity of detecting sputum *lytA* by PCR were 94% and 96%, respectively (21), while Albrich et al. reported these values to be 77.8% and 66.7%, respectively (22). The low accuracy observed in the study of Albrich et al. (22) can be explained by two reasons. First, the characteristics of the patients were different in the two studies. The study of Stralin et al. (21) comprised 78 elderly patients (mean age, 78 years), while the study of Albrich et al. (22) comprised 222 HIV-infected young patients (mean age, 36 years). Second, the two studies used different combinations of diagnostic tests as their reference; the study of Stralin et al. (21) used a combination of blood cultures, sputum cultures, and ICT, while the study of Albrich et al. (22) used a combination of blood cultures, whole-blood *lytA* PCR, ICT, and sputum cultures. The current study recruited community-dwelling elderly patients (mean age, 77 years) and used sputum cultures and ICT as references, which was similar to the study of Stralin et al. (21). Therefore, the high accuracy of using qPCR to detect *lytA* observed in our study may be compatible with that in the study of Stralin et al. (21).

This study has some limitations. First, we did not have blood samples for culture from all of the participants. According to our previous study (19), only 14 of 1,310 cases (1%) were positive for pneumococcus by blood culturing. We also estimated the blood culture-positive rates to be quite low in this study population, so we presume that the low rates of blood culture positivity are not problematic. Second, we included all of the obtained sputum samples upon admission or upon the first visit to the outpatient department at the study site regardless of sputum quality. Some participants, especially the elderly patients, could not expectorate sputum of good quality in this clinical situation. However, we expected that the use of pneumococcal DNA loads with previously established cutoff values can discriminate infection from upper respiratory tract colonization regardless of sputum quality (21, 22). To verify this speculation, we analyzed the accuracy of the nanofluidic Sp-qPCR with only sputum samples that were Geckler group 3 or more (39). In BLCMs with 186 quality-controlled nanofluidic Sp-qPCR assays, the sensitivity and specificity did not differ much from those obtained using all sputum samples (data not shown). This finding may suggest that the quality of sputum does not affect the accuracy of nanofluidic Sp-qPCR assays; however, further studies are needed to establish the diagnostic value of sputum quality. Third, some nonpneumococcal streptococci which contain genes similar to pneumococcal capsule genes might have coexisted in our sputum samples and might have been detected by our serotype-specific PCR assays (40, 41). This detection might have increased the frequency of the observed multiple pneumococcal serotypes. However, we screened all samples by PCR for the *lytA* gene to identify the presence of pneumococcus and serotyped only *lytA*-positive samples. Moreover, our *in silico* analyses confirmed that none of our primer sequences used for pneumococcal serotyping amplified nonpneumococcal streptococci. We therefore believe that the potential impact of the existence of nonpneumococcal streptococci on our accuracy estimates was minimal.

In conclusion, the nanofluidic Sp-qPCR assay may be highly accurate and useful for detecting VTPP among adults. Further studies are needed to establish the clinical implication of the detection of multiple serotypes by the nanofluidic Sp-qPCR assay and the discrepancies between the nanofluidic Sp-qPCR and other diagnostic methods.

## Supplementary Material

Supplemental material
