# Supplementary material for: Accuracy of High-Throughput Nanofluidic PCR-Based Pneumococcal Serotyping and Quantification Assays Using Sputum Samples for Diagnosing Vaccine Serotype Pneumococcal Pneumonia: Analyses by Composite Diagnostic Standards and Bayesian Latent Class Models
Source: J Clin Microbiol. 2018 Apr 25;56(5):e01874-17. doi: 10.1128/JCM.01874-17 (PMC5925721; doi:10.1128/JCM.01874-17)
Supplement: Supplemental material [file supp_56_5_e01874-17__index.html]

Accuracy of High-Throughput Nanofluidic PCR-Based Pneumococcal Serotyping and Quantification Assays Using Sputum Samples for Diagnosing Vaccine Serotype Pneumococcal Pneumonia: Analyses by Composite Diagnostic Standards and Bayesian Latent Class Models — Supplemental material 

# Accuracy of High-Throughput Nanofluidic PCR-Based Pneumococcal Serotyping and Quantification Assays Using Sputum Samples for Diagnosing Vaccine Serotype Pneumococcal Pneumonia: Analyses by Composite Diagnostic Standards and Bayesian Latent Class Models

## Supplemental material

- Supplemental file 1 -

  Table S1 (Pneumococcal positivity status by diagnostic test and cutoff value)

  PDF, 277K
- Supplemental file 2 -

  Table S2 (Vaccine serotype pneumococcal positivity status by diagnostic test and cutoff value)

  PDF, 133K
- Supplemental file 3 -

  Table S3 (Serotype results determined by serotype-specific qPCR, culturing, and UAD)

  PDF, 149K
- Supplemental file 4 -

  Table S4 (Estimated disease prevalence, sensitivity, and specificity of qPCR for *lytA* using sputum samples for diagnosing pneumococcal pneumonia)

  PDF, 469K
- Supplemental file 5 -

  Table S5 (Estimated disease prevalence, sensitivity, and specificity of qPCR for *lytA* using sputum samples, sputum cultures, and urinary antigen tests for diagnosing pneumococcal pneumonia)

  PDF, 125K
- Supplemental file 6 -

  Fig. S1 (Patient selection flow chart)

  PDF, 131K
- Supplemental file 7 -

  Fig. S2 (Serotype-specific DNA load by serotype-specific urinary antigen detection assay result among patients in whom serotype-specific DNA is detectable)

  PDF, 817K
