# Supplementary material for: Accuracy of High-Throughput Nanofluidic PCR-Based Pneumococcal Serotyping and Quantification Assays Using Sputum Samples for Diagnosing Vaccine Serotype Pneumococcal Pneumonia: Analyses by Composite Diagnostic Standards and Bayesian Latent Class Models
Source: J Clin Microbiol. 2018 Apr 25;56(5):e01874-17. doi: 10.1128/JCM.01874-17 (PMC5925721; doi:10.1128/JCM.01874-17)
Supplement: Supplemental material [file JCM.01874-17_zjm999095916s1.pdf]

Supplementary table 1. Pneumococcal positive status by diagnostic tests and cutoff values (n=244)

**All pneumococcus**	**Positive, n (%)**	**Antibiotics Prescription, n (%)***		**P-value****
		yes	no	
**Immunochromatographic test**	30 (12.3)	6 (10.0)	24 (13.0)	0.534
**Sputum culture, cutoff value (CFU/ml)**				
**10^4**	28 (11.5)	6 (10.0)	22 (12.0)	0.680
**10^5**	26 (10.7)	6 (10.0)	20 (10.9)	0.850
**10^6**	23 (9.4)	4 (6.7)	19 (10.3)	0.401
**10^7**	18 (7.4)	3 (5.0)	15 (8.2)	0.418
**10^8**	8 (3.3)	2 (3.3)	6 (3.3)	0.978
**qPCR for *lytA*, cutoff value (DNA copies/ml)**				
**10^3**	53 (21.7)	12 (20.0)	41 (22.3)	0.710
**10^4**	46 (18.9)	11 (18.3)	35 (19.0)	0.906
**10^5**	42 (17.2)	11 (18.3)	31 (16.9)	0.792
**10^6**	37 (15.2)	9 (15.0)	28 (15.2)	0.968
**10^7**	28 (11.5)	5 (8.3)	23 (12.5)	0.380
**10^8**	14 (5.7)	5 (8.3)	9 (4.9)	0.321
**10^9**	6 (2.5)	2 (3.3)	4 (2.2)	0.615

qPCR: quantitative PCR, *: diagnostics positive rate is based on antibiotics prescribed group (n=60) and not antibiotics prescribed group (n=184), **: Wilcoxon rank-sum test of diagnostics positive rate between antibiotics prescribed group and not antibiotics prescribed group
