# Supplementary material for: Accuracy of High-Throughput Nanofluidic PCR-Based Pneumococcal Serotyping and Quantification Assays Using Sputum Samples for Diagnosing Vaccine Serotype Pneumococcal Pneumonia: Analyses by Composite Diagnostic Standards and Bayesian Latent Class Models
Source: J Clin Microbiol. 2018 Apr 25;56(5):e01874-17. doi: 10.1128/JCM.01874-17 (PMC5925721; doi:10.1128/JCM.01874-17)
Supplement: Supplemental material [file JCM.01874-17_zjm999095916s2.pdf]

Supplementary table 2. Vaccine serotype pneumococcal positive status by diagnostic tests and cutoff values (n=244)

**PCV13 serotypes**	**Positive, n (%)**	**Antibiotics Prescription, n (%)***		**P-value****
		yes	no	
**Serotype-specific urinary antigen detection**	27 (11.1)	7 (11.7)	20 (10.9)	0.865
**Sputum culture and quellung reaction, cutoff value (CFU/ml)**				
**10^4**	16 (6.6)	4 (6.7)	12 (6.5)	0.969
**10^5**	14 (5.7)	4 (6.7)	10 (5.4)	0.722
**10^6**	12 (4.9)	3 (5.0)	9 (4.9)	0.973
**10^7**	10 (4.1)	3 (5.0)	7 (3.8)	0.686
**10^8**	6 (2.5)	2 (3.3)	4 (2.2)	0.615
**Serotype-specific qPCR, cutoff value (DNA copies/ml)**				
**10^3**	36 (14.8)	10 (16.7)	26 (14.1)	0.631
**10^4**	34 (13.9)	10 (16.7)	24 (13.0)	0.483
**10^5**	30 (12.3)	8 (13.3)	22 (12.0)	0.778
**10^6**	24 (9.8)	6 (10.0)	18 (9.8)	0.961
**10^7**	21 (8.6)	5 (8.3)	16 (8.7)	0.931
**10^8**	16 (6.6)	5 (8.3)	11 (6.0)	0.523
**10^9**	8 (3.3)	5 (8.3)	3 (1.6)	0.012

PCV13: 13-valent pneumococcal polysaccharide conjugate vaccine, qPCR: quantitative PCR, *: diagnostics positive rate is based on antibiotics prescribed group (n=60) and not antibiotics prescribed group (n=184), **: Wilcoxon rank-sum test of diagnostics positive rate between antibiotics prescribed group and not antibiotics prescribed group
