# Supplementary material for: Accuracy of High-Throughput Nanofluidic PCR-Based Pneumococcal Serotyping and Quantification Assays Using Sputum Samples for Diagnosing Vaccine Serotype Pneumococcal Pneumonia: Analyses by Composite Diagnostic Standards and Bayesian Latent Class Models
Source: J Clin Microbiol. 2018 Apr 25;56(5):e01874-17. doi: 10.1128/JCM.01874-17 (PMC5925721; doi:10.1128/JCM.01874-17)
Supplement: Supplemental material [file JCM.01874-17_zjm999095916s3.pdf]

Supplementary Table 3. Serotype results determined by serotype-specific qPCR, culturing and UAD

ID	Serotype-specific qPCR (DNA copies/mL)			Sputum culture (CFU/mL)	UAD
	1st serotype	2nd serotype	3rd serotype		
1	3(10^8)	10A(10^7)	35F(10^3)	3(10^6)	3
2	10A(10^7)	3(10^6)	35F(10^4)	neg	3
3	10A(10^7)	4(10^7)	17F(10^6)	neg	4
4	3(10^8)	17F(10^5)	18(10^5)	3(10^8)	3
5	4(10^5)	34(10^5)	10A(10^4)	neg	neg
6	10A(10^6)	35B(10^5)	5(10^5)	neg	neg
7	4(10^8)	10A(10^7)	17F(10^6)	4(10^8)	4
8	10A(10^5)	35B(10^5)	9N/9L(10^2)	neg	neg
9	19A(10^9)	9N/9L(10^5)	8(10^4)	19A(10^7)	19A
10	10A(10^8)	22F/A(10^7)	35F(10^5)	neg	neg
11	19A(10^9)	12F(10^5)	9N/9L(10^5)	19A(10^8)	19A
12	10A(10^9)	5(10^6)	-	10A(10^7)	neg
13	11(10^9)	35B(10^4)	-	11A(10^6)	neg
14	10A(10^9)	4(10^4)	-	10A(10^8)	neg
15	19A(10^9)	5(10^4)	-	19A(10^8)	19A
16	6(10^9)	9N/9L(10^6)	-	NG (10^8)	6
17	10A(10^6)	5(10^5)	-	NG (10^7)	neg
18	3(10^5)	6(10^4)	-	3(10^7)	neg
19	23A(10^8)	4(10^3)	-	neg	neg

20	10A(10^6)	6(10^4)	-	neg	6
21	6(10^6)	18(10^4)	-	neg	6, 18
22	22F/A(10^8)	3(10^4)	-	neg	neg
23	19A(10^9)	4(10^3)	-	neg	neg
24	3(10^9)	10A(10^6)	-	3(10^8)	3
25	22F/A(10^8)	4(10^4)	-	22F(10^5)	23F
26	3(10^9)	-	-	3(10^4)	neg
27	3(10^8)	-	-	3(10^4)	3
28	3(10^8)	-	-	3(10^5)	3
29	3(10^7)	-	-	3(10^7)	neg
30	3(10^7)	-	-	neg	3
31	3(10^5)	-	-	neg	3
32	4(10^3)	-	-	11A(10^7)	neg
33	11(10^5)	-	-	neg	neg
34	34(10^6)	-	-	neg	neg
35	10A(10^5)	-	-	neg	neg
36	15B/C(10^8)	-	-	neg	neg
37	19A(10^9)	-	-	19A(10^5)	19A
38	19A(10^7)	-	-	19A(10^7)	neg
39	19F(10^5)	-	-	neg	neg
40	22F/A(10^8)	-	-	no data(10^7)	neg
41	23F(10^8)	-	-	neg	23F
42	33F/33A/37(10^7)	-	-	neg	neg
43	35B(10^9)	-	-	35B(10^7)	neg

44	6(10^7)	-	-	neg	6A
45	6(10^8)	-	-	6B(10^6)	6B
46	6(10^8)	-	-	6B(10^8)	6B
47	NT	-	-	neg	neg
48	NT	-	-	NG (10^6)	neg
49	NT	-	-	neg	neg
50	NT	-	-	15A(10^6)	neg
51	NT	-	-	neg	neg
52	NT	-	-	neg	23F
53	NT	-	-	neg	neg
54	-	-	-	neg	23F
55	-	-	-	neg	1
56	-	-	-	neg	23F
57	-	-	-	neg	1

NT: non typeable; minus(-): no serotypes were detected in serotype-specific qPCR column; neg: negative results in sputum culture and UAD column; NG: no growth (serotype was not detected because we could not subculture at our laboratory); no data: strain lost at our laboratory; qPCR: quantitative PCR; UAD: serotype-specific urinary antigen detection assay
