# Supplementary material for: Accuracy of High-Throughput Nanofluidic PCR-Based Pneumococcal Serotyping and Quantification Assays Using Sputum Samples for Diagnosing Vaccine Serotype Pneumococcal Pneumonia: Analyses by Composite Diagnostic Standards and Bayesian Latent Class Models
Source: J Clin Microbiol. 2018 Apr 25;56(5):e01874-17. doi: 10.1128/JCM.01874-17 (PMC5925721; doi:10.1128/JCM.01874-17)
Supplement: Supplemental material [file JCM.01874-17_zjm999095916s4.pdf]

Supplementary table 4. Estimated disease prevalence, sensitivity and specificity of qPCR for *lytA* using sputum sample for diagnosing pneumococcal pneumonia (cutoff value 1.0×10^4 DNA copies/ml)

**Reference standard**	**Immunochromatographic test**			**Immnochromatographic test + sputum culture***		
	Disease prevalence** (95% CI)	Sensitivity (95% CI)	Specificity (95% CI)	Disease prevalence** (95% CI)	Sensitivity (95% CI)	Specificity (95% CI)
	12.3%	83.3%	90.2%	16.8%	85.4%	94.6%
	(8.5-17.1)	(65.3-94.4)	(85.4-93.8)	(12.3-22.1)	(70.8-94.4)	(90.5-97.3)

* conventional sputum culture and quellung reaction's bacterial load cutoff value was 1.0×10^5 CFU/ml, ** estimated from reference standard or composite reference standard result assumed 100% specificity, qPCR: quantitative PCR, CI: confidence interval
