# Supplementary material for: Accuracy of High-Throughput Nanofluidic PCR-Based Pneumococcal Serotyping and Quantification Assays Using Sputum Samples for Diagnosing Vaccine Serotype Pneumococcal Pneumonia: Analyses by Composite Diagnostic Standards and Bayesian Latent Class Models
Source: J Clin Microbiol. 2018 Apr 25;56(5):e01874-17. doi: 10.1128/JCM.01874-17 (PMC5925721; doi:10.1128/JCM.01874-17)
Supplement: Supplemental material [file JCM.01874-17_zjm999095916s5.pdf]

Supplementary table 5. Estimated disease prevalence, sensitivity and specificity of qPCR for *lytA* using sputum sample, sputum culture, and urinary antigen test for diagnosing pneumococcal pneumonia: Bayesian latent class model analysis

lytA qPCR, cutoff value (copies/ml)	**Disease prevalence (95%CrI)**	**Sputum *lytA* PCR**		**Sputum culture***		**ICT**		
		Sensitivity (95% CrI)	Specificity (95% CrI)	Sensitivity (95% CrI)	Specificity (95% CrI)	Sensitivity (95% CrI)	Specificity (95% CrI)	Youden Index of PCR
$\geq 10^3$	18.8% (12.6-26.1)	91.4% (74.6-99.7)	94.0% (88.6-99.1)	55.5% (37.3-74.2)	99.0% (97.1-99.9)	58.4% (40.8-76.0)	97.7% (94.5-99.8)	0.854
$\geq 10^4$	18.3% (12.6-24.9)	91.4% (74.5-99.8)	97.0% (92.7-99.8)	56.7% (39.4-74.2)	99.1% (97.3-99.9)	60.0% (43.2-76.2)	97.8% (94.8-99.8)	0.884
$\geq 10^5$	18.1% (12.4-24.8)	86.2% (67.8-98.6)	97.5% (93.8-99.9)	57.5% (40.0-75.3)	99.1% (97.3-100)	59.9% (43.4-76.1)	97.6% (94.3-99.8)	0.837
$\geq 10^6$	17.0% (11.7-23.4)	85.0% (64.7-98.5)	98.6% (95.8-100)	61.0% (42.7-78.2)	99.1% (97.2-100)	61.7% (45.1-77.3)	97.3% (93.8-99.8)	0.836
$\geq 10^7$	16.2% (10.5-23.5)	68.6% (46.3-89.0)	98.8% (96.5-100)	64.1% (42.7-84.8)	99.0% (96.9-100)	61.3% (43.8-77.8)	96.6% (92.6-99.8)	0.674
$\geq 10^8$	10.3% (5.2-17.7)	54.5% (27.7-82.4)	99.1% (97.3-100)	74.7% (43.2-98.0)	95.9% (92.4-98.8)	78.1% (53.9-95.4)	94.8% (90.2-99.4)	0.536
$\geq 10^9$	11.4% (5.6-19.5)	24.4% (9.1-46.8)	99.5% (98.0-100)	71.1% (42.3-96.6)	96.2% (92.3-99.5)	76.8% (49.7-97.1)	95.4% (90.7-99.6)	0.239

* bacterial load cutoff value was 10^5 CFU/ml, qPCR: quantitative PCR, CrI: credible interval, ICT: immunochromatographic test
