# Supplementary material for: Accuracy of High-Throughput Nanofluidic PCR-Based Pneumococcal Serotyping and Quantification Assays Using Sputum Samples for Diagnosing Vaccine Serotype Pneumococcal Pneumonia: Analyses by Composite Diagnostic Standards and Bayesian Latent Class Models
Source: J Clin Microbiol. 2018 Apr 25;56(5):e01874-17. doi: 10.1128/JCM.01874-17 (PMC5925721; doi:10.1128/JCM.01874-17)
Supplement: Supplemental material [file JCM.01874-17_zjm999095916s7.pdf]

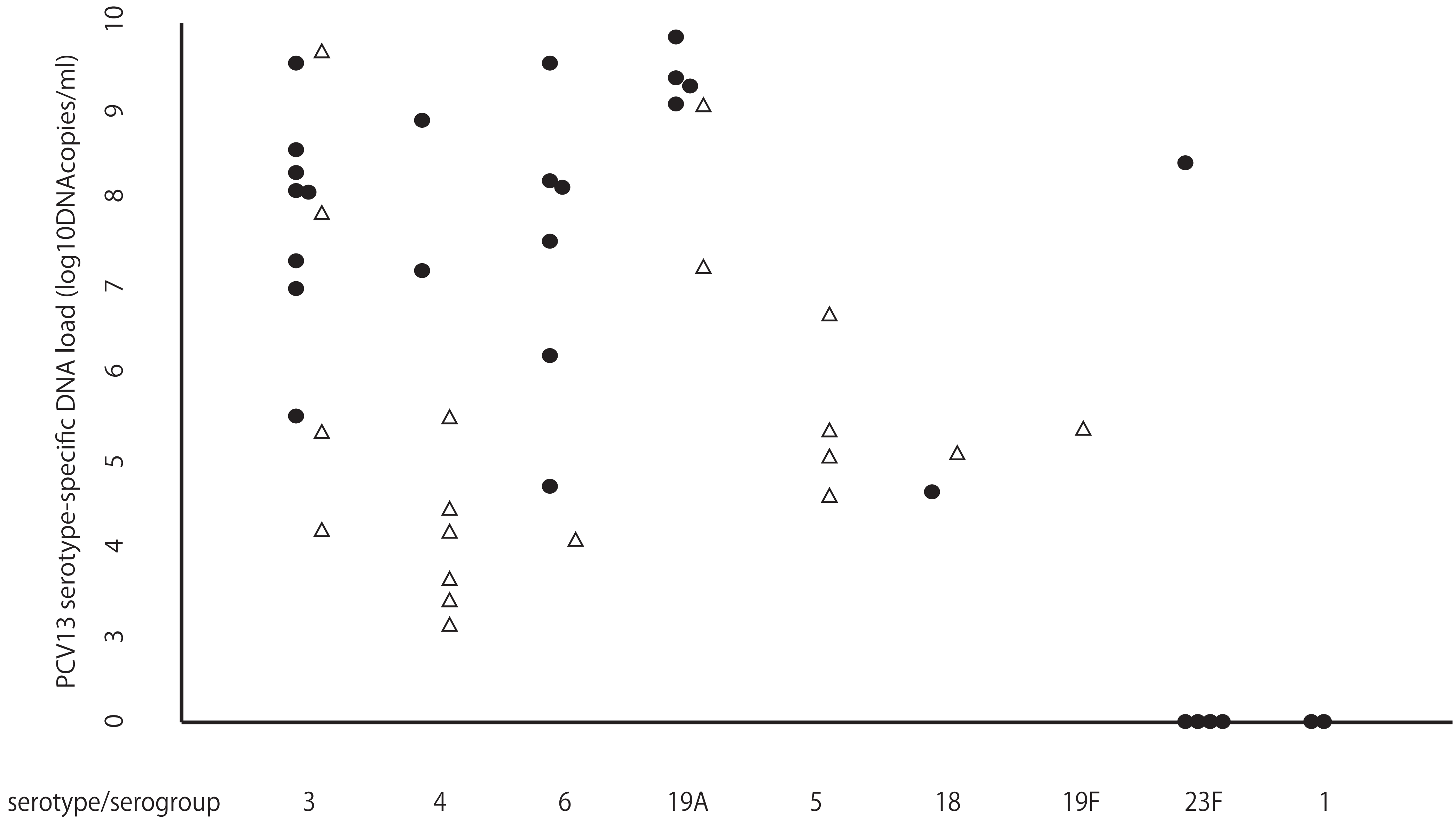

Supplementary figure 2. Serotype-specific DNA load by serotype-specific urinary antigen detection assay results among serotype-specific DNA detectable patients
circle: UAD positive case, pyramidal shape: UAD negative case, PCV13: 13-valent pneumococcal polysaccharide conjugate vaccine
